# Exploration of ligand binding modes towards the identification of compounds targeting HuR: a combined STD-NMR and Molecular Modelling approach

**DOI:** 10.1038/s41598-018-32084-z

**Published:** 2018-09-13

**Authors:** Francesca Vasile, Serena Della Volpe, Francesca Alessandra Ambrosio, Giosuè Costa, M. Yagiz Unver, Chiara Zucal, Daniela Rossi, Emanuela Martino, Alessandro Provenzani, Anna K. H. Hirsch, Stefano Alcaro, Donatella Potenza, Simona Collina

**Affiliations:** 1 Department of Chemistry, University of Milan, Via Golgi 19, 20133 Milano, Italy; 2 Department of Drug Sciences, Medicinal Chemistry and Technology Section, University of Pavia, Via Taramelli 12, 27100 Pavia, Italy; 3 Department of Health Sciences, University “Magna Græcia” of Catanzaro, Viale Europa, 88100 Catanzaro, Italy; 4 Helmholtz Institute for Pharmaceutical Research Saarland (HIPS) - Helmholtz Centre for Infection Research (HZI), Department of Drug Design and Optimization, Campus building E8.1, 66123 Saarbrücken, Germany; 5 Centre for Integrative Biology, CIBIO, University of Trento, Via Sommarive 9, 38123 Povo, TN Italy; 6 Department of Earth and Environmental Sciences, University of Pavia, via S. Epifanio 14, 27100 Pavia, Italy; 7 Department of Pharmacy, Medicinal Chemistry, Saarland University, Campus building E8.1, 66123 Saarbrücken, Germany

## Abstract

Post-transcriptional processes have been recognised as pivotal in the control of gene expression, and impairments in RNA processing are reported in several pathologies (i.e., cancer and neurodegeneration). Focusing on RNA-binding proteins (RBPs), the involvement of Embryonic Lethal Abnormal Vision (ELAV) or Hu proteins and their complexes with target mRNAs in the aetiology of various dysfunctions, has suggested the great potential of compounds able to interfere with the complex stability as an innovative pharmacological strategy for the treatment of numerous diseases. Here, we present a rational follow-up investigation of the interaction between ELAV isoform HuR and structurally-related compounds (*i.e.*, flavonoids and coumarins), naturally decorated with different functional groups, by means of STD-NMR and Molecular Modelling. Our results represent the foundation for the development of potent and selective ligands able to interfere with ELAV–RNA complexes.

## Introduction

Post-transcriptional modifications have a crucial role in regulating gene expression by shaping the fate of RNA transcripts in their journey from the nucleus (i.e., alternative splicing, poly-adenylation, nuclear export) to the ribosome (i.e., cytoplasmic localisation, stability, translation rate). Dysfunctions within these routes may be directly involved in several pathologies, such as neurodegenerative diseases, inflammation and cancer^1–5^.

In this context, RNA-binding proteins (RBPs), have been recognised to be play a prominent role as they affect the fate of target messenger RNAs (mRNAs) coding for proteins pivotal in key cellular functions^1–5^.

**Figure 1:**
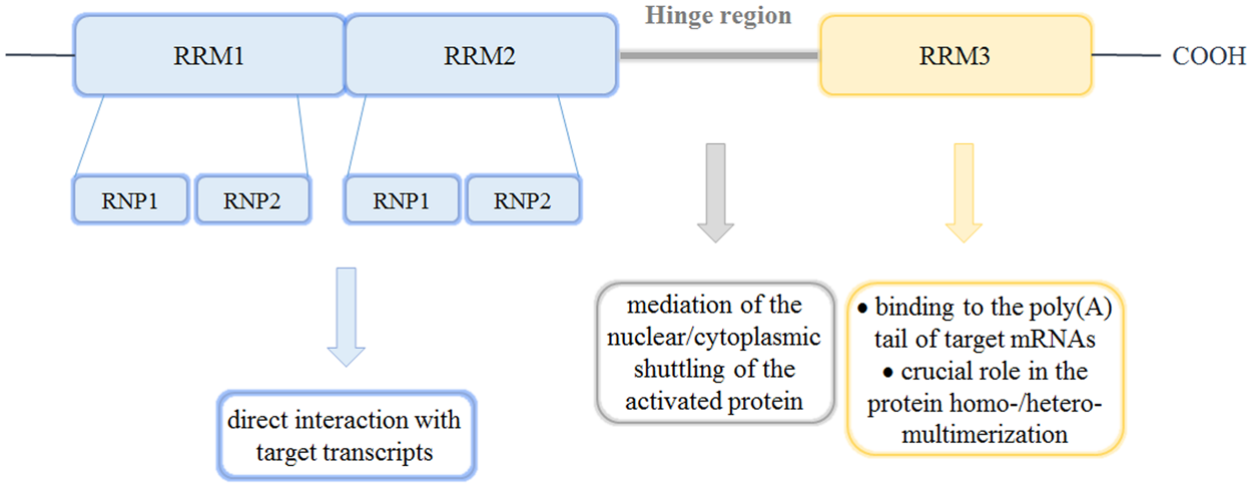
ELAV domains and their involvement in protein function.

Embryonic Lethal Abnormal Vision (ELAV) or Hu proteins are among the better characterised RBPs with 4 human mammalian isoforms: ubiquitous HuR, and HuB, HuC and HuD prevalently expressed in the nervous system (nELAVs)^6^. These proteins share a high degree of sequence homology (70–85%): they contain three RNA recognition motif-type (RRM) domains, each approximately 90 amino-acid in length^7^; the first two consecutive domains (RRM1 and RRM2) are near the N-terminus and link to the third domain (RRM3) by an unconserved hinge region, responsible for the nuclear/cytoplasmic shuttling occurring after protein activation^8–10^. RRM1 and RRM2 directly interact with target transcripts through highly conserved ribonucleoprotein (RNP) sequences 1 and 2, formed by 8 and 6 amino acids, respectively^11,12^ while RRM3, aside from binding the poly(A) tail of target mRNAs, has a crucial role in the homo- and heteromultimerisation of ELAV proteins (Fig. 1)^13–15^.

The interaction of the four mammalian ELAV proteins with many mRNAs, gives rise to various ELAV protein−RNA complexes, characterised by different physiological roles. As a consequence, ELAV proteins have a potential as pharmacological targets in several pathologies and compounds able to interfere with ELAV–RNA complexes may lead to different effects.

Particularly, it has been demonstrated that the RNA-binding protein HuR is highly abundant in many cancers and it may either be a marker for malignancy or have an oncogenic role in numerous tumor systems including breast, ovarian, and colon^16–18^. In fact, numerous HuR‐regulated mRNAs encode proteins implicated in carcinogenesis^19^. For this reason, HuR can be considered a promising candidate target for governing gene regulatory mechanisms. Specifically, it was shown that HuR is upregulated and dysregulated in cancer cells, in part, through post-transcriptional gene regulation^19,20^. Among the small natural products that have been reported to inhibit HuR function, it is worth mentioning MS-444, since it has been shown to prevent translocation of HuR (and its associated mRNA cargos) to the cytoplasm. In a number of tumor cells, HuR inhibition by MS-444 leads to a dose-dependent reduction in cell proliferation by promoting apoptosis^21^. The majority of compounds interacting/interfering with HuR−RNA complexes have been discovered by screening a large number (or a library) of commercially available compounds with high-throughput screening (HTS) approaches and using various biological assays based on different detection technologies^21,22^. Among them, dihydrotanshinone (DHTS), which is a nanomolar disruptor of HuR–RNA binding^23^, has potent HuR-dependent antitumor activity *in vivo* and is able to inhibit HuR multimerization^24,25^. Starting from the DHTS scaffold, medicinal-chemistry efforts led to the discovery of a series of small molecules called Tanshinone Mimics (TMs) with improved affinity and potency compared to DHTS^26^. Summing up, and going beyond the state of the art of HuR modulators available so far, the identification of the key druggable pockets on the surface of HuR is the essential milestone for discovering new molecular scaffolds.

As a part of our ongoing efforts in this field, we had already demonstrated the importance of the four RNP sequences in binding and stabilising a target transcript RNA by means of real-time quantitative PCR^27^, molecular modelling^28^ and advanced NMR techniques (Saturation-Transfer Difference, STD, and Diffusion-Ordered Spectroscopy, DOSY)^29^.

In the present work, we set out to study the interaction between HuR and a number of compounds performing a systematic study which combines a ligand-based NMR technique, namely STD-NMR, with a molecular modelling study. It is well known that STD-NMR can be used as an epitope mapping device to describe the target–ligand interactions^30–32^ and it can be applied to weak and transient protein–ligand complexes that are difficult to study by other structural methods^33,34^. We compared results of STD-NMR with molecular dynamics and docking simulations, thus affording an improved understanding of ligand–HuR interactions. Despite the high protein mobility and width of the protein–RNA interface, the combination of NMR and *in silico* studies resulted to be a reliable tool for understanding HuR-ligand binding modes^35,36^. The exploitation of these interactions lays the foundation for the design of *ad hoc* molecules endowed with HuR–RNA complex interfering properties.

## Results

### Selection of compounds, solubility and stability assessment

**Figure 2:**
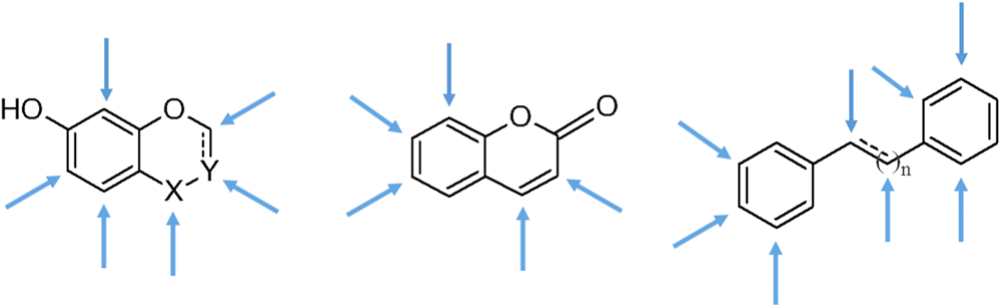
General structures of the naturally-occurring compounds selected; the arrows define the possible positions of decoration.

**Figure 3:**
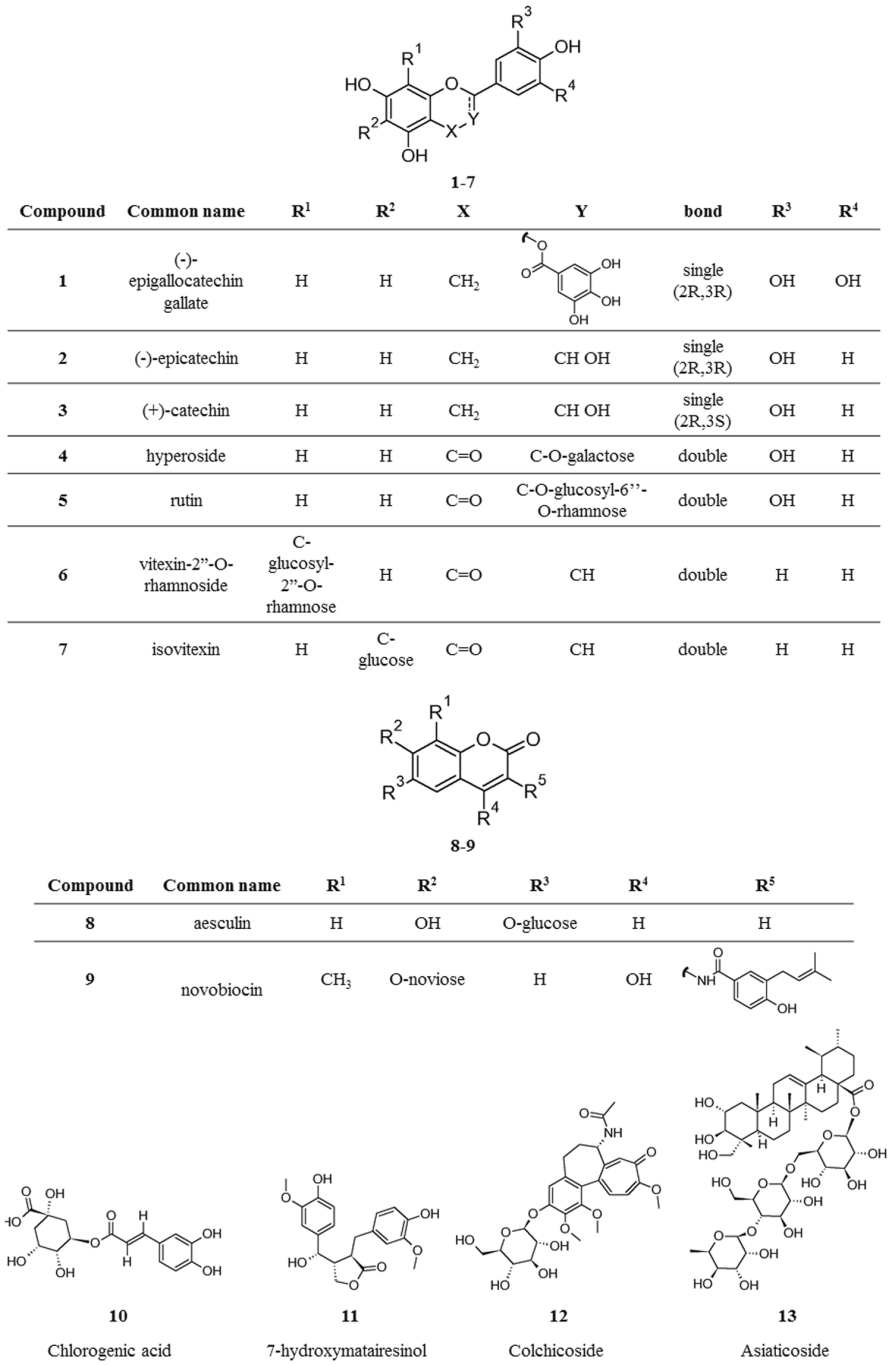
Structures of the naturally occurring products ultimately subjected to the study.

Our previous docking results had revealed that a series of natural products, *i.e.* epigallogatechin gallate, quercetin, okicenone, myricetyn, DHTS, MS-444 and others (described in SI), interact with HuR in the same regions as the target RNAs, more specifically with the RNP1 and RNP2 sequences of the RRM1 and RRM2 domains of the protein^22^. Based on these preliminary data, with the aim to study the ligand-HuR interaction, we collected a small series of compounds of natural origin, namely flavones, flavonols, flavan-3-ols and coumarins, naturally decorated with different functional groups, as well as some unrelated compounds with similar features and a high degree of structural diversity (Figs 2, 3). We then tested their solubility and stability in the buffer and time-ranges required by STD-NMR experiments (24–48 hours). In detail, a 1 mM solution of each compound was dissolved in a 20 mM deuterated phosphate buffer within a 5.0–7.4 pH range (where necessary, a DMSO-d6 percentage ≤10% was added), at a 283–303 K temperature range and studied by preliminary ^1^H-NMR experiments.

Among the tested molecules, we selected 13 soluble and stable compounds (Fig. 3) and used them in STD-NMR experiments to explore the ligand-protein interaction mode.

### NMR interaction studies with HuR

Prior to the interaction study with HuR, ^1^H, COSY, TOCSY, HSQC and NOESY spectra of the selected compounds were recorded in the proper deuterated phosphate buffer solution (pH range 5.8–7.4) at 298 K and 283 K. The assignment of all compounds is reported in SI.

STD-NMR is one of the most widespread NMR techniques used to study the interactions between small ligands and macromolecules^37,38^. This method is based on the transfer of saturation from the protein to the bound ligand which in turn, by exchange, is moved into solution where it is detected. During the period of saturation (saturation time), the magnetisation gradually moves from the protein to the protons of the ligand when it binds to the target. The saturation time is chosen taking into account the efficiency of saturation transfer from protein protons during the bound state (intermolecular protein–ligand NOEs) and the rate of accumulation of saturated ligand molecules in the free state. Long saturation times permit to map all ligand contacts. This saturation process is very efficient, so the modulation of the ligand signal induced by the protein is readily detected, even in the presence of a large excess of ligand. The ligand protons nearest to the protein are most likely to be saturated to the highest degree, and therefore have the strongest signal in the one-dimensional STD spectrum. The ligand protons located further away are saturated to a lower degree, and their STD intensities are weaker. Therefore, the degree of saturation of individual ligand protons (expressed as absolute-STD %) reflects their proximity to the protein surface and can be used as an epitope-mapping method to describe the target–ligand interactions^39,40^. STD-NMR spectra were initially acquired with varying saturation times from 0.98 s to 2.94 s and using different ligand–protein ratios (from about 500:1 to 1000:1). This large molar excess of ligand was employed in order to preclude the perturbations of absolute STD intensities due to rebinding effects, which would impede the correct determination of the group epitope mapping. Additionally, negative controls were performed to avoid artefacts due to the presence of signals in the blank. The optimal conditions found entail the use of 2.94 s, with high ligand/protein ratios (around 1000:1) for all compounds and exploiting the Watergate sequence for water suppression.

The absolute STD percentages of each ligand protons were quantified in order to analyse their proximity to the protein surface (short protein–ligand distances produce a strong intensity of the corresponding STD signal). On the other hand, also relative STD percentages were calculated for each compound, by normalising all measured STD intensities against the most intense signal (which is arbitrarily assigned a value of 100%). The obtained group epitope mapping then illustrates which chemical moieties of the ligand are key for molecular recognition in the binding site.

STD-NMR spectra were acquired and processed accordingly for all 13 compounds and showed that 12 out of the 13 compounds studied interact with HuR displaying different intensities and epitopes of interaction. Given the difficulty of determining *K*_D_ values for each compound with STD-NMR experiments due to the compounds poor solubility, we analysed the absolute STD data following two different keys: (a) the intensity of the STD signals (indicative of the proximity to the protein) and (b) the number of interactions for each ligand.

By reporting the number of the signals and related intensity of each compound, Table 1 gives a picture of their interaction with target HuR. Most of the compounds studied are characterised by a weak interaction with the protein within the absolute STD range of 0.4***–***0.1% and only a few ligands present STD signals exceeding the unit value (Table 1). Remarkably, compound **9** gives the highest number of interactions, while **6** is characterised by fewer and weaker interactions with the protein.

**Table 1:** Compound and related number of interacting protons within listed absolute STD range.

Compound	Number of interacting protons					
	*Absolute STD % ranges*					
	>*1*	*1.0–0.9*	*0.8–0.7*	*0.6–0.5*	*0.4–0.2*	<*0.2*
1			2			4
2				2	3	2
3				2	1	5
4			1		3	1
5	3				5	1
6					1	6
7		1			2	2
8					2	2
9	2	2	1	1	7	
10	1			1	5	
11			1	2	5	5
12	1		2	1	3	
13	no signal shown					

Moreover, compound **5** has the highest number of very strong interactions and several of medium and low intensity. On the other hand, the STD-NMR spectra for **13** and HuR showed absence of signal, indicative of lack of interaction between the two entities.

**Figure 4:**
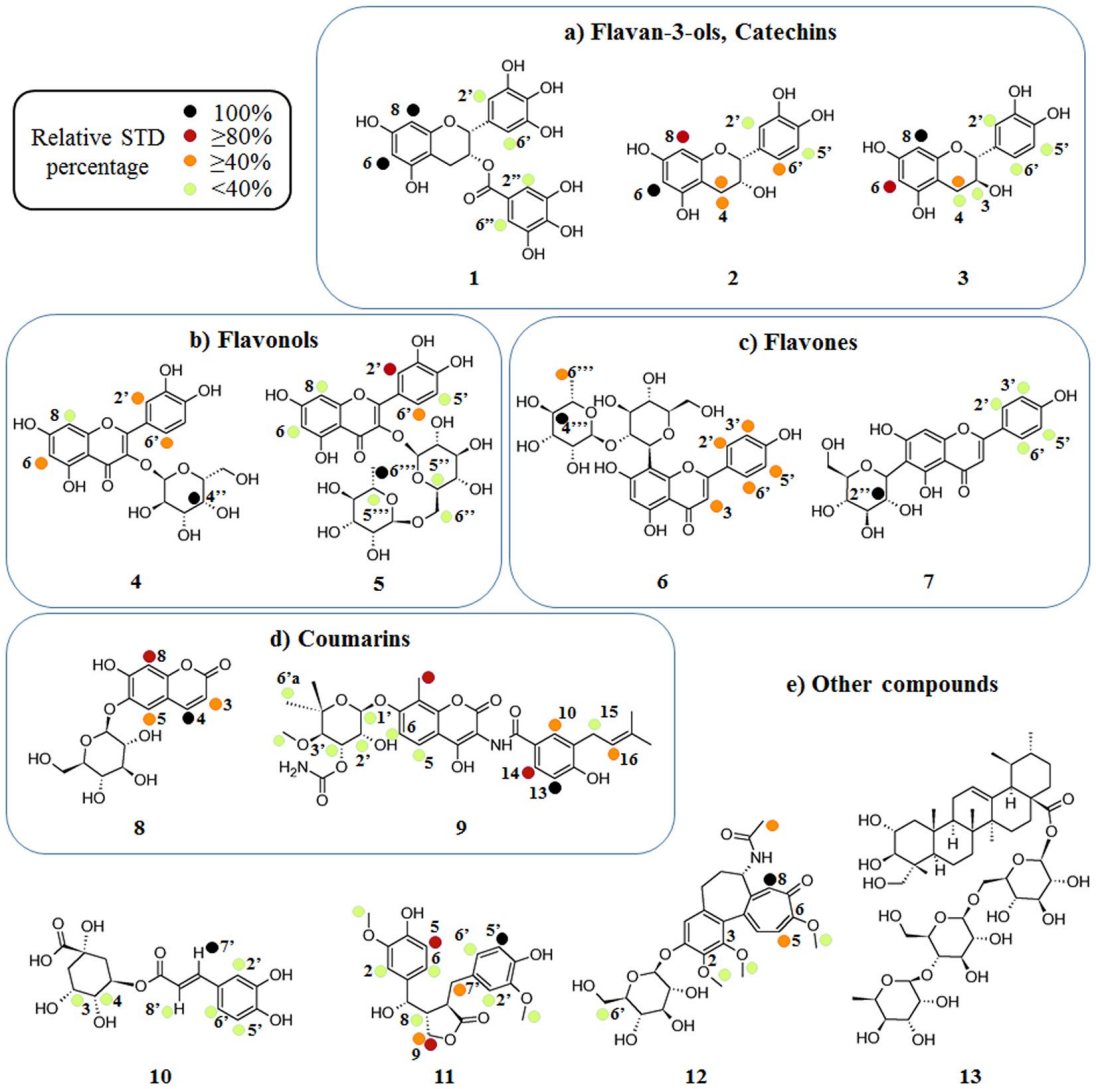
Group epitope mapping is highlighted for each compound. Relative STD percentages are conveyed by colour code: black dots indicate the most intense signal (100% relative STD), dark red dots over 80%, orange dots over 40%, and lime green dots under 40% relative to the most intense STD signal.

**Figure 5:**
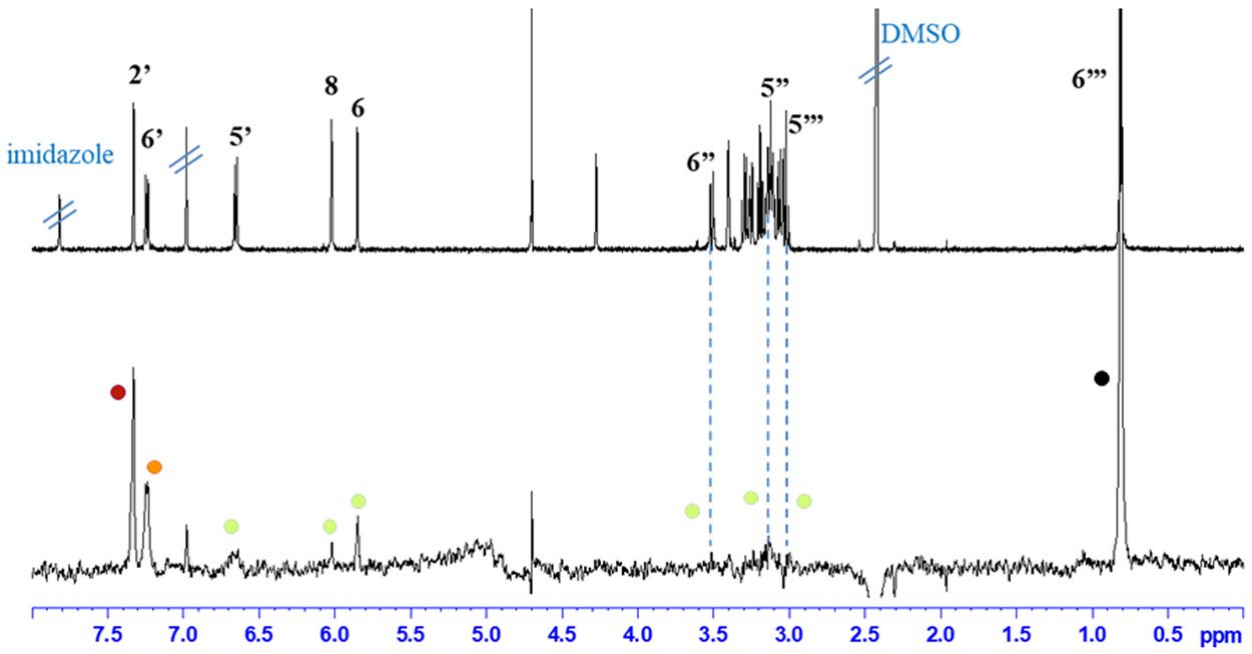
^1^H and STD spectrum of compound **5**; coloured dots convey relative STD % according to the following colour code, black dots indicate the most intense signal arbitrarily assigned the value of 100% relative STD; dark red dots represent relative STD over 80%, orange dots over 40%, and lime green dots under 40%; all relative to the most intense STD signal.

In addition to absolute STD% to evaluate the binding epitope of each compound, we calculated relative STD% as shown in Fig. 4 conveyed by colour code; black dots are used to indicate the most intense signal arbitrarily assigned the value of 100% relative STD. Subsequently, dark red dots represent relative STD over 80%, orange dots over 40%, and lime green dots under 40%; all are relative to the most intense STD signal. Additionally, in Fig. 5, we report the ^1^H and STD spectra for compound **5** and the corresponding coloured dots for relative STD% according to the colour code just described.

### *In silico* studies on HuR protein

For the preliminary selection of the compounds to be used in our study, we utilised the rigid docking approach previously published^22^, which allowed us to quickly evaluate their potential. In order to better characterise the results obtained through STD-NMR, we decided to exploit a complementary *in silico* approach, which is computationally more demanding but enables a more detailed description of the ligand–protein behaviour in solution.

**Figure 6:**
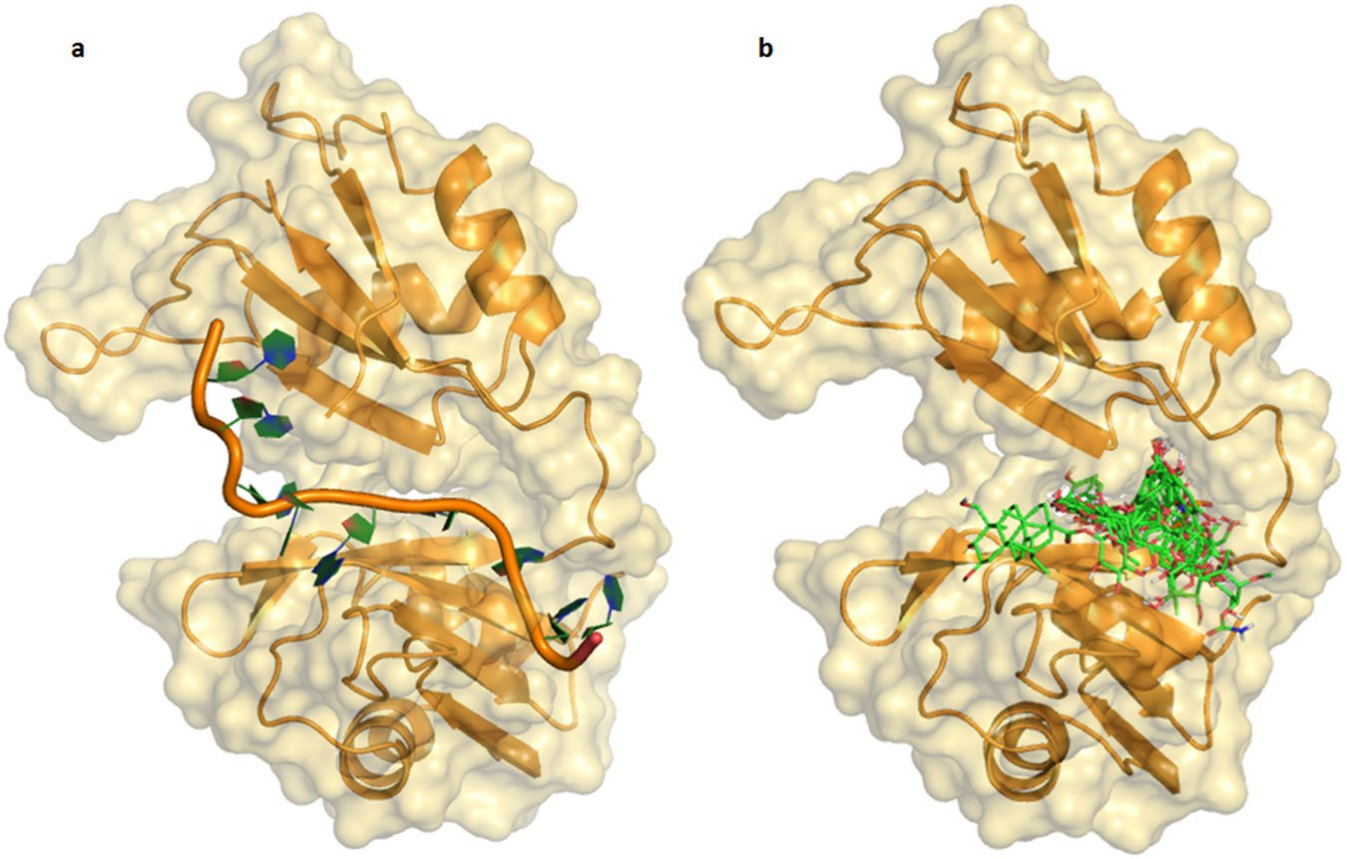
(**a**) 3D representation of HuR RRM1 and RRM2 domain–RNA^*c-fos*^ complex in its “closed” conformation; (**b**) Superimposition of all studied compounds in the “closed” conformation of the HuR–RNA interaction site. HuR and RNA^*c-fos*^ are represented as light-orange and orange cartoon, respectively; all compounds are depicted as green sticks.

**Figure 7:**
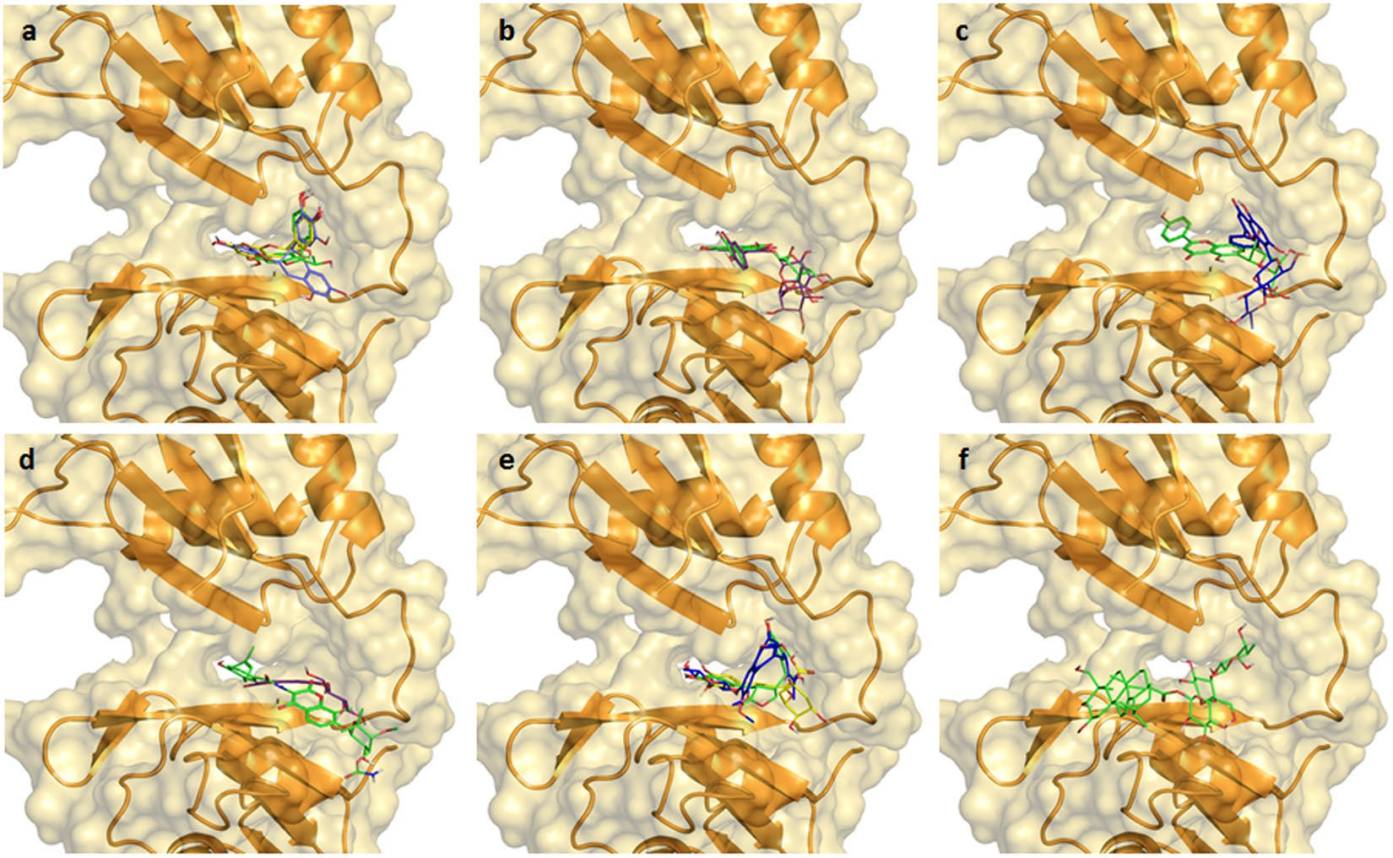
3D representation of compounds divided by compound class in the HuR–RNA interaction site: (**a**) compounds **1**, **2**, **3** (blue, green and yellow sticks, respectively); (**b**) compounds **4** and **5** (green and violet sticks respectively); (**c**) compounds **6** and **7** (blue and green, respectively); (**d**) compounds **8** and **9** (violet and green sticks); (**e**) compounds **10**, **11** and **12** (yellow, green, blue sticks, respectively); (**f**) compound **13** shown as green sticks; HuR protein shown as orange cartoon^60^.

The protein crystal structure represents one of the many possible substates of the protein and, in most cases, the overall topology of the folded state is conserved, but a different orientation of even a single side chain in the binding site can significantly influence docking results^41^. Thus, in order to consider all conformational states of HuR and to investigate potential protein rearrangement, we submitted the HuR RRM domain–RNA^*c-fos*^ complex (PDB code: 4ED5) to 500 ns of Molecular Dynamics simulations (MDs)^42^. The result trajectory was clustered with respect to the Root Mean Square Deviation (RMSD), affording ten representative structures, which we used for the following docking studies. By applying this approach, we were able to reproduce the results previously reported on the two main conformational states of the HuR RRM domains, “open” and “closed”^24,43^. We analysed how the ligands can bind the two states and we observed that the STD contacts better fit with the “closed” state of HuR. Here, we will describe the interaction of each ligand with a HuR snapshot corresponding to the “closed” conformation. Molecular-recognition studies revealed that 12 of the 13 considered compounds interact with the same region in the HuR interaction site (Figs 6b and 7), in a deep pocket of domains RRM1 and RRM2, while compound **13** appears to interact with their external surface (docking poses divided by class of compound are reported in Fig. 7, while poses for single compounds are reported in SI). All contacts between the HuR binding site and ligands will be identified and catalogued in terms of hydrophobic contacts, hydrogen bonds and cation-π interactions.

Furthermore, it is worth noting that the HuR “closed” form thus explained could exceed the sole descriptive purpose and be exploited as a model for future structure-based virtual screening (SBVS) studies using different compound libraries, as already implemented on different targets in our previous investigations^44,45^, in order to identify new ligands interfering with the HuR–RNA complex.

## Discussion

STD-NMR and molecular modelling results have been compared to gain a more detailed picture of the ligand–protein interactions. As previously mentioned, MD simulations of HuR clearly showed that the protein can exist in two prevalent structural conformations: the “open” conformation and the “closed” one; the comparison of experimental STD-NMR and theoretical docking results, led us to hypothesise that the “closed” conformation is predominant in presence of interacting ligands.

For simplicity purposes, we present all the following considerations in the next section as a comparison of the results divided by class of compound. Combined NMR and docking data for all interacting compounds are reported in supporting information, where for each compound, the intensity of the STD signal of each proton is compared with the number of the interactions observed *in silico*.

Concerning Flavan-3-ols or catechins (**1**–**3**), the three compounds considered differ only in the substitution and stereochemistry of the carbon atom in position 3; in particular, **2** and **3** only differ in the configuration of carbon 3 (respectively, (*R*) and (*S*)), while **1** (*R*) hosts an additional aromatic ring. STD experiments showed that the protons in positions 6 and 8 of the chroman-3-ol nucleus (structures in Figs 4a and SI) are mainly involved in their interaction with HuR. For all compounds, the aromatic nucleus in position 2, and the additional aromatic ring (gallate ester) in position 3 for compound **1**, participate in the interaction, even though to a lesser degree. Docking results show that all compounds interact in the same region of the HuR interaction site (Fig. 7a); in particular, we observed that all aromatic rings in position 2 overlap and are involved in a cation-π interaction with Arg97. On the other hand, the chroman-3-ol nucleus of **1** does not show overlapping interactions with the same nucleus of compounds **2** and **3**, as it projects towards Phe65, establishing hydrophobic interactions, whereas, the chroman-3-ol nucleus of compounds **2** and **3** overlaps with the gallate ester moiety of **1**. Moreover, considering the evidence that **2** and **3** show very similar interactions, it is fair to assume that, within their scaffold, the absolute configuration at carbon 3 does not play a relevant role in the interaction with target HuR.

Within the flavonol compound class (**4** and **5**), the chromone-3-ol nucleus and the aromatic ring in position 2 are highlighted as interacting regions (Figs 4b and SI), in line with the STD results obtained with the previous class (Flavan-3-ols). Furthermore, in both cases, the additional sugar rings in position 3 reinforce the interaction with the protein. *In silico* results show compounds **4** and **5** bind to HuR by interacting with the same residues (Arg153, Tyr63, Arg97, Pro98). However, the second sugar moiety of compound **5** (rhamnose) establishes additional hydrophobic interactions with Lys55 and Ile52 and a hydrogen bond with Tyr63 of RRM1, justifying a slight deviation from the binding site for compound **5** (Fig. 7b); this is also in accordance with the strong STD signals seen for this sugar moiety.

Different to the first two classes of flavonoids, the chromone nucleus of Flavones (6 and 7) shows almost complete absence of STD peaks (Figs 4c and SI), which denotes a scarce contact with the protein, while, albeit weak, the interaction of the phenols in position 2 is still observed. The sugar moieties interact with the target protein HuR with different intensities, namely 1% absolute STD for the proton in position 2″ of **7**, and 0.25% absolute STD for 4′′′ of **6**. Accordingly, **6** and **7** show different binding poses and very limited common interactions (Fig. 7c), as compound **7** elongates in the site forming hydrogen bonds with Asn82, Asn 25, Arg97 and Pro98, while compound **6** adopts a rather closed conformation and interacts with Arg153 and Ile103 of RRM2 and Tyr63 and Lys55 of RRM1.

Focusing on coumarins (**8** and **9**), within STD experiments, compound **8** displayed few and weak interactions with HuR, presumably due to its small size. Furthermore, the sugar ring in position 6 does not improve its contact with HuR (Figs 4d and SI). In contrast, the STD spectrum of **9** showed a large number of intense peaks relative to both the coumarin nucleus and the conjugated moiety on the right-hand side (Figs 4d and SI). Moreover, the STD peaks relative to the noviose moiety, although weaker, convey its contribution to increasing the interaction with the target HuR. Accordingly, docking studies reveal compounds **8** and **9** fit differently into the HuR interaction site, most likely due to their different size (Fig. 7d). In particular, the unsaturated conjugated regions of **9** protrude all along the considered interaction site, establishing hydrophobic interactions with Ile132 and Ile133 of RRM2 and Lys92, Val93 and Ser94 of RRM1 and hydrogen bonds with Arg153 of RRM2 and Asn25 of RRM1 with the sugar portion projecting towards the solvent, providing additional interactions with Lys55 of RRM1.

As mentioned above, compounds **10**–**13** (Fig. 4e) are structurally different to both the original ones and the others involved in the study. For all of them, STD-NMR spectra show that the main interactions are achieved through the presence of unsaturated conjugated regions. Related docking results show compounds **10**–**12** occupy the deep part of the interaction site (Fig. 7e), as they appear to be involved in hydrogen-bond interactions with amino acids lining the deep part of the pocket such as Arg97, Arg131, Arg153, Asp105.

Finally, the STD spectrum of compound **13** in presence of HuR shows no signals indicating the absence of interactions. Within docking studies against the “closed” conformation of the protein, compound **13** only appears to interact with the external surface of the HuR–RNA interacting site probably due to steric hindrance, and does not overlap with the same region interacting with all other molecules. As a consequence, the *in vitro* interaction may be too weak to fall in the detection range of STD-NMR.

In addition to the considerations reported and discussed inside each compound class, it is worth remarking that recurring patterns can be recognised within the entirety of the compound collection investigated or wider subgroups.

Particularly, among flavonoid derivatives, flavan-3-ols and flavonols (Figs 4a,b, 7a,b and SI), both STD-NMR epitopes and *in silico* data show similar data, while flavones (Figs 4c, 7c and SI) show different behaviours. This has been attributed to both the difference in substitution positions (position 3 for the first two classes, versus 6 and 8 for flavones) and in sugar linking (O- and C-glycoside, respectively), which increase the rigidity of the flavone scaffold.

Moving on to comment on the whole library, the importance of unsaturated conjugated regions as well as aromatic rings in the interaction with HuR has been noted throughout both STD-NMR and molecular modelling; additionally, these moieties seem to fit in repeated regions, and thus show hydrophobic interactions with repeated residues, namely Ile23, Asn25, Ser94, Ile133 and Arg153. Moreover, concerning the sugar moiety counterparts, we observed that the sugar rings directly attached to the aromatic ring of compounds **4**, **5**, **6**, **7** and **8** and the quinic acid moiety of **10**, overlap in the same region of the HuR binding site, establishing contacts with repeated residues (Arg97, Pro98, Ser99 and Ile 103).

To sum up, the results we obtained thus far evidenced that all interacting compounds interact with repeated residues of RNP1 and RNP2 of RRM1 domain; compounds **1**–**12** interact deep in the interaction site, while **13** protrudes more outwardly towards the solvent. Furthermore, our study corroborates the already reported observation that interacting compounds could stabilise a closed conformation of the HuR binding site^22,26,46^.

## Conclusions

The acknowledgement of the crucial role of post-transcriptional processes in the control of gene expression and their connection to various diseases has opened up a new fascinating route for discovering new drugs. RNA binding proteins, and particularly those belonging to the ELAV family, can affect the fate of target mRNAs whose coded proteins are fundamental for key cellular functions^22,47^. Interfering compounds may specifically affect the fate of ELAV protein−mRNA complexes at various levels, in both the nucleus and the cytoplasm, and also depending on the transcript targeted, as well as on other modulating factors (i.e., other RBPs and miRNAs). Since compounds interfering with all ELAV proteins may give rise to several effects, the tailored design and tissue-targeted delivery of selective compounds will be essential. To date, little structural information are available and a rational drug-design approach is difficult to employ at this stage and this is still considered a challenging research. In this manuscript, by focussing on HuR, owing to its high potential in cancer therapy and diagnosis^48,49^ and in line with this observation, we studied the molecular recognition between natural ligands and the protein at the atomic level. We followed a systematic medicinal-chemistry approach based on structural biophysical studies by STD-NMR combined with molecular modelling. To properly explore the chemical space, we collected and selected a library of compounds of natural origin, applying structural diversity criteria.

The combined STD-NMR/molecular modelling approach allowed us to study the direct interactions between HuR and small molecules. By defining the ligand epitope of interaction and corroborating useful aspects about the protein conformational changes and HuR–ligand interactions, our findings represent a pivotal starting point to drive a drug discovery program. The preliminary SAR considerations drawn in this work will drive the design of a new *ad hoc* small focused compound library. In the near future, our efforts will be directed along this long and winding road.

## Methods

### Preliminary docking studies

The preliminary docking investigation on all 28 selected compounds was carried out as already described^22^.

### Characterisation of the natural compounds, solubility and stability assessment

To assess the compound stability and solubility, ^1^H-NMR spectra were acquired on a 400 MHz Bruker Avance spectrometer using a 1 mM solution of each compound in a 20 mM deuterated phosphate buffer within a 5.0–7.4 pH range, and a 283–303 K temperature range; all occurring peak variations due to instability or solubility issues were monitored over time within a 48 h time period by acquiring ^1^H spectra at regular intervals; a DMSO-d6 percentage ≤10% was allowed to dissolve the less soluble compounds.

The full compound characterisation at the optimised conditions (pH 5.8 or 7.4, DMSO-d6% and 283 K) was performed for the final 13 selected compounds and required the further acquisition and assignment of COSY, TOCSY, HSQC and NOESY spectra.

### Protein expression and purification

Protein expression, purification and purity assessment for HuR aliquots utilised in the STD-NMR study were performed as already described^23,50^.

### STD-NMR

All protein/ligand samples were prepared in a 1000:1 ligand/protein ratio. Typically, the final concentration of the samples was 400 μM of ligand and 0.4 μM of HuR, and the final volume was 500 μL. The buffer used is a 20 μM deuterated phosphate buffer with 10% H_2_O, pH 5.8 for compounds **1**–**3**, **9**, **10**; pH 7.4 for **4**–**8**, **11**–**13**; an additional amount of DMSO-d6 ≤ 10% was used to aid the solubility of compounds **4**, **5**, **7**, **11** and **13**.

^1^H–STD-NMR experiments were performed at 600 MHz on a Bruker Avance spectrometer. The probe temperature was maintained at 283 K. In the STD experiments, water suppression was achieved by WATERGATE 3–9–19 pulse sequence. The on-resonance irradiation of the protein was performed at a chemical shift of −0.05 ppm for all compounds with the exception of compound **13** which was irradiated at –2.00 ppm to avoid artefacts. Off-resonance irradiation was applied at 200 ppm, where no protein signals are visible. Selective presaturation of the protein was achieved by a train of Gauss-shaped pulses of 49 ms length each. The STD-NMR spectra were acquired with varying saturation times from 0.98 s to 2.94 s; the optimised total length of the saturation train was 2.94 s for all compounds except for compound **1** for which we analysed the STD experiment at 0.98 s due to its instability observed after longer experimental timeframes.

Intensities of all STD effects (absolute STD) were calculated by division through integrals over the respective signals in STD-NMR reference spectra. The different signal intensities of the individual protons are best analysed from the integral values in the reference and STD spectra, respectively. (I_0_ − I_sat_)/I_0_ is the fractional STD effect, expressing the signal intensity in the STD spectrum as a fraction of the intensity of an unsaturated reference spectrum. In this equation, I_0_ is the intensity of one signal in the off-resonance or reference NMR spectrum, I_sat_ is the intensity of a signal in the on-resonance NMR spectrum, and I_0_ − I_sat_ represents the intensity of the STD-NMR spectrum.

### Molecular Dynamics and Docking simulations

Starting from the crystal structure of the two N-terminal RRM domains of HuR complexed with RNA, deposited in the Protein Data Bank (PDB) with 4ED5 PDB code^51^, we performed our modelling simulations. The HuR-RNA complex was prepared through Protein Preparation Wizard implemented in Maestro using OLPS-2005 as force field^52–54^. Residual crystallographic buffer components and water molecules were removed, missing side chains were built using the Prime module^55^, hydrogen atoms were added, side chains protonation states at pH 7.4 were assigned. The structure was then submitted to 10000 of MacroModel minimisation steps using OPLS-2005 as force field^54,56^. Molecular Dynamics (MD) simulations were run using Desmond package v. 3.8 at 300 K temperature and ensamble NPT class^57,58^. The system was immersed in an orthorhombic box of TIP4P water molecules, extending at least 10 Å from the protein, and counter ions were added to neutralise the system charge. The resulting trajectory was clustered with respect to Root Mean Square Deviation (RMSD), in order to explore all the collection structures obtained, getting ten representative structures, which were submitted to 10.000 MacroModel minimisation steps, using OLPS-2005 as force field. Docking studies were carried out with Glide, software by using SP v. 6.7 (standard precision) algorithm and the binding pocket was identified by placing a cube centred on the mRNA, 10 poses for ligand were generated^59^.

## Electronic supplementary material

Supplementary Information
