# Supplementary material for: Exploration of ligand binding modes towards the identification of compounds targeting HuR: a combined STD-NMR and Molecular Modelling approach
Source: Sci Rep. 2018 Sep 13;8:13780. doi: 10.1038/s41598-018-32084-z (PMC6137155; doi:10.1038/s41598-018-32084-z)
Supplement: Supplementary file 1 — Supplementary Information [file 41598_2018_32084_MOESM1_ESM.docx]

**Exploration of ligand binding modes towards the identification of compounds targeting HuR: a combined STD-NMR and Molecular Modeling approach**

Francesca Vasile,^1#^ Serena Della Volpe,^1,2#^ Francesca Alessandra Ambrosio,^3^ Giosuè Costa,^3^ M. Yagiz Unver,^4^ Chiara Zucal,^5^ Daniela Rossi,^2^ Emanuela Martino,^6^ Alessandro Provenzani,^5^ Anna K. H. Hirsch,^4,7^ Stefano Alcaro,^3^ Donatella Potenza^1^ and Simona Collina^2^

^1^Department of Chemistry, University of Milan, Via Golgi 19, 20133 Milano, Italy

^2^Department of Drug Sciences, Medicinal Chemistry and Technology Section, University of Pavia, Via Taramelli 12, 27100 Pavia, Italy

^3^Department of Health Sciences, University “Magna Græcia” of Catanzaro, Viale Europa, 88100 Catanzaro, Italy

^4^Helmholtz Institute for Pharmaceutical Research Saarland (HIPS) - Helmholtz Centre for Infection Research (HZI), Department of Drug Design and Optimization, Campus building E8.1, 66123 Saarbrücken, Germany

^5^Centre for Integrative Biology, CIBIO, University of Trento, Trento 38122, Italy

^6^Department of Earth and Environmental Sciences, University of Pavia, I- 27100 Pavia, Italy;

^7^Department of Pharmacy, Medicinal Chemistry, Saarland University, Campus building E8.1, 66123 Saarbrücken, Germany

**Supporting Information**

1. **Chemical structure of compounds interfering with ELAV protein–RNA complexes** S2
2. **NMR spectra assignment for all compounds (283K)** S3
3. **Predicted binding modes for all compounds** S8
4. **Combined STD-NMR and Docking histograms for all interacting compounds** S9
5. **Chemical structure of compounds interfering with ELAV protein–RNA complexes**

**
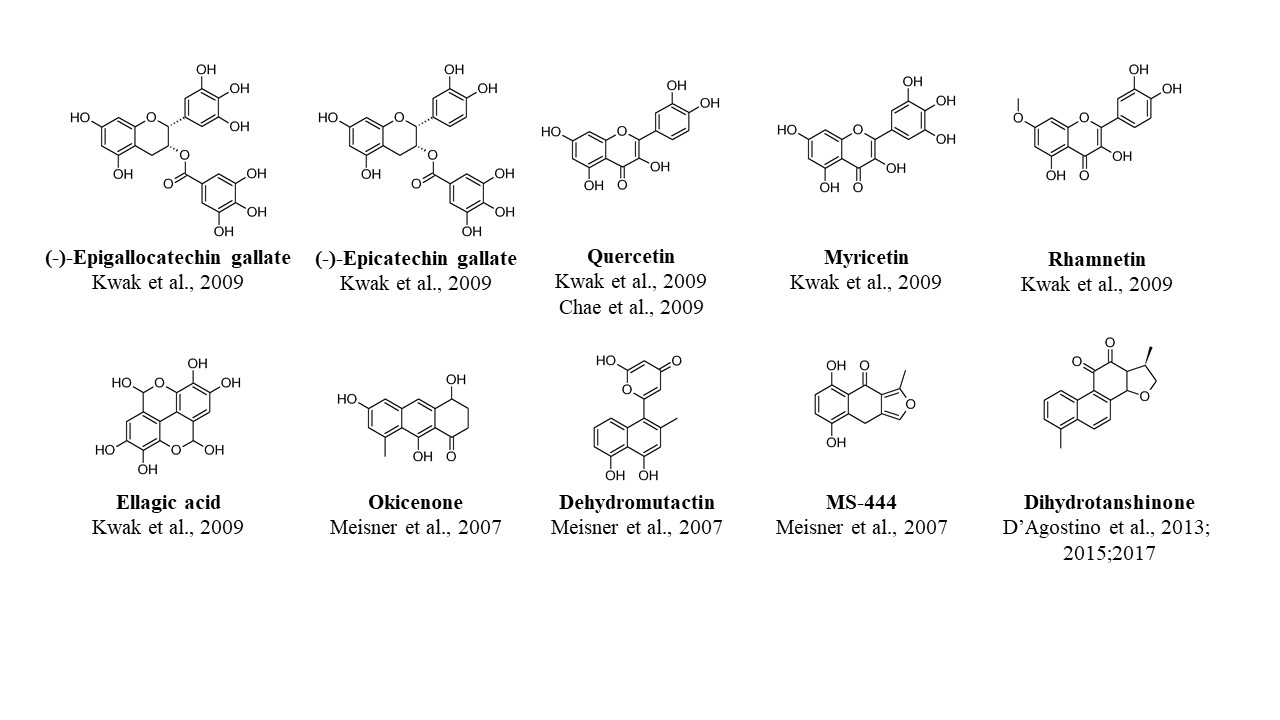
**

Epigallocatechin gallate, Epicatechin gallate, Quercetin, Myricetin, Rhamnetin and Ellagic acid were identified as inhibitors of the complex HuC–artificial ARE sequences.

Okicenone, Dehydromutacin and MS-444 were isolated from microbial extracts and identified as the first HuR protein inhibitors.

Dihydrotanshinone was described as inhibitor of the formation of HuR–RNA complex.

1. **NMR spectra assignment for all compounds (283K)**

| δH | ^13^C |  |
| --- | --- | --- |
| 6.73 | 109.70 | 2’’-6’’ |
| 6.34 | 106.27 | 2’-6’ |
| 5.89 | 95.44 | 6-8 |
| 5.36 | 68.50 | 3 |
| 4.80 | 76.69 | 2 |
| 2.80 | 24.40 | 4 |
| 2.68 |  |  |

| δH | ^13^C |  |
| --- | --- | --- |
| 6.78 | 114.51 | 6’ |
| 6.68 | 119.05 | 2’ |
|  | 116.21 | 5’ |
| 5.85 | 95.53 | 8 |
| 5.83 | 95.84 | 6 |
| 4.74 | 78.70 | 2 |
| 4.06 | 66.20 | 3 |
| 2.67 | 27.60 | 4 |
| 2.52 |  |  |

| δH | ^13^C |  |
| --- | --- | --- |
| 6.67 | 114.66 | 2’ |
|  | 116.66 | 5’ |
| 6.60 | 119.69 | 6’ |
| 5.84 | 95.43 | 8 |
| 5.75 | 94.54 | 6 |
| 4.55 | 80.50 | 2 |
| 3.96 | 66.17 | 3 |
| 2.62 | 25.56 | 4 |
| 2.27 |  |  |

| δH | ^13^C |  |
| --- | --- | --- |
| 7.39 | 116.51 | 6’ |
| 7.28 | 122.47 | 2’ |
| 6.30 | 115.85 | 5’ |
| 6.03 | 95.84 | 8 |
| 5.85 | 101.40 | 6 |
| 4.63 | 102.77 | 1’’ |
| 3.60 | 67.76 | 4’’ |
| 3.51 | 71.00 | 2’’ |
| 3.33 | 72.18 | 3’’ |
| 3.32 | 59.80 | 6’’ |
| 3.23 | 75.13 | 5’’ |

**^^**

| δH | ^13^C |  |
| --- | --- | --- |
| 7.33 | 116.5 | 2’ |
| 7.24 | 122.76 | 6’ |
| 6.65 | 115.38 | 5’ |
| 6.02 | 95.53 | 8 |
| 5.85 | 101.60 | 6 |
| 4.68 | 102.09 | 1’’ |
| 4.28 | 100.62 | 1’’’ |
| 3.51 | 67.57 | 6’’ |
| 3.41 | 69.92 | 2’’’ |
| 3.29 | 73.53 | 2’’ |
| 3.25 | 69.92 | 3’’’ |
| 3.20 | 75.60 | 3’’ |
| 3.14 | 68.89 | 5’’ |
| 3.12 | 74.73 | 4’’’ |
| 3.06 | 69.74 | 4’’ |
| 3.02 | 71.81 | 5’’’ |
| 0.82 | 16.52 | 6’’’ |

**^^**

| δH | ^13^C |  |
| --- | --- | --- |
| 7.69 | 128.69 | 2’-6’ |
| 6.75 | 115.70 | 3’-5’ |
| 6.32 | 102.22 | 3 |
| 5.89 | 101.00 | 6 |
| 4.81 | 72.11 | 1’’ |
| 4.75 | 100.22 | 1’’’ |
| 4.00 | 76.20 | 2’’ |
| 3.66 | 69.57 | 2’’’ |
| 3.51 | 71.20 | 5’’ |
| 3.48 | 79.23 | 3’’ |
| 3.39 | 61.81 | 6’’ |
| 3.29 |  |  |
| 3.32 | 79.89 | 4’’ |
| 3.24 | 69.09 | 3’’’ |
| 2.86 | 71.00 | 4’’’ |
| 2.02 | 68.44 | 5’’’ |
| 0.40 | 16.53 | 6’’’ |

**^^**

| δH | ^13^C |  |
| --- | --- | --- |
| 7.54 | 128.47 | 2’-6’ |
| 6.64 | 116.43 | 3’-5’ |
| 6.23 | 102.20 | 3 |
| 6.10 | 96.90 | 6 |
| 4.61 | 73.74 | 1’’ |
| 4.16 | 70.16 | 2’’ |
| 3.64 | 60.83 | 6’’ |
| 3.54 |  |  |
| 3.32 | 69.76 | 3’’ |
| 3.30 | 78.10 | 4’’ |
| 3.28 | 80.48 | 5’’ |

**^^**

| δH | ^13^C |  |
| --- | --- | --- |
| 7.61 | 146.50 | 4 |
| 6.98 | 113.22 | 5 |
| 6.42 | 104.38 | 8 |
| 5.88 | 107.49 | 3 |
| 4.77 | 101.4 | 1’ |
| 3.70 | 60.44 | 6’ |
| 3.52 |  |  |
| 3.40 | 75.71 | 2’ |
| 3.38 | 73.20 | 4’ |
| 3.35 | 72.88 | 5’ |
| 3.25 | 69.19 | 3’ |

**^^**

| δH | ^13^C |  |
| --- | --- | --- |
| 7.50 | 121.0 | 10 |
| 7.47 | nd | 5 |
| 7.44 | nd | 13 |
| 6.89 | 108.8 | 6 |
| 6.70 | 114.6 | 14 |
| 5.43 | 96.1 | 1 |
| 5.12 | nd | 16 |
| 5.09 | 70.5 | 4’ |
| 4.07 | 68.0 | 2’ |
| 3.35 | 80.7 | 3’ |
| 3.31 | 60.3 | OCH_3_ |
| 3.08 | 27.4 | 15 |
| 2.03 | nd | CH_3_ |
| 1.47 | 24.5 | 17-18 |
| 1.17 | 26.6 | 6’a |
| 0.95 | 22.2 | 6’b |

**^^**

| δH | ^13^C |  |
| --- | --- | --- |
| 7.40 | 146.10 | 7’ |
| 6.93 | 114.81 | 2’ |
| 6.85 | 122.49 | 6’ |
| 6.66 | 116.28 | 5’ |
| 6.13 | 114.82 | 8’ |
| 5.06 | 70.90 | 5 |
| 3.97 | 70.32 | 3 |
| 3.61 | 72.51 | 2 |
| 1.93 | 37.70 | 6 |
| 1.80 |  |  |
| 1.85 | 37.19 | 4 |
| 1.75 |  |  |

**^^**

| δH | ^13^C |  |
| --- | --- | --- |
| 6.47 | 115.01 | 5 |
| 6.41 | 115.30 | 5’ |
| 6.37 | 117.74 | 6 |
| 6.27 | 109.33 | 2 |
| 6.23 | 122.03 | 2’ |
| 6.20 | 112.23 | 6’ |
| 4.54 | 73.72 |  |
| 4.23 | 71.3 | 9 |
| 4.14 |  |  |
| 3.41 | 55.15 | OCH_3_-OCH_3_’ |
| 2.64 | 34.94 | 7’ |
| 2.33 |  |  |
| 2.50 | 42.35 | 8 |
| 2.40 | 44.94 | 8’ |

**^^**

| δH | ^13^C |  |
| --- | --- | --- |
| 7.26 | 137.50 | 5 |
| 7.20 | 130.00 | 8 |
| 7.05 | 115.94 | 4 |
| 6.65 | 111.60 | 12 |
| 4.95 | 100.07 | 1’ |
| 4.17 | 53.11 |  |
| 3.71 | 56.71 | 6-OCH_3_ |
| 3.70 | 60.64 | 6’ |
| 3.49 |  |  |
| 3.68 | 61.8 | 2-OCH_3_ |
| 3.36-3.43 | 72.99-75.95 | Overlap, 2’,3’,4’ |
| 3.34 | 61.62 | 3-OCH_3_ |
| 3.26 | 69.51 | 5’ |
| 2.32 | 28.72 | 10 |
| 1.68 |  |  |
| 2.00 | 35.29 | 11 |
| 1.93 |  |  |
| 1.77 | 21.90 | 9-NHCO*CH_3_* |

**-CH:**

| δH | ^13^C |  |
| --- | --- | --- |
| 5.32 | 94.04 | A1 |
| 5.22 | 125.52 | 12 |
| 4.79 | 101.00 | R1 |
| 4.47 | 101.97 | B1 |
| 3.95 | 69.13 | R5 |
| 3.89 | 70.41 | R2 |
| 3.74 | 68.62 | 2 |
| 3.66 | 70.24 | R3 |
| 3.63 | 76.48 | A3 |
| 3.48 | 77.28 | *n.d.* |
| 3.47 | 75.69 | *n.d.* |
| 3.48 | 74.73 | *n.d.* |
| 3.43 | 68.95 | A |
| 3.40 | 71.87 | R4 |
| 3.34 | 75.17 | *n.d.* |
| 3.30 | 76.66 | *n.d.* |
| 3.21 | 73.44 | B2 |
| 2.09 | 52.64 | 18 |
| 1.57 | 47.07 | 9 |
| 1.36 | 38.56 | 20 |
| 1.14 | 46.70 | 5 |
| 0.90 | 38.60 | 19 |

**-CH_2_:**

| δH | ^13^C |  |
| --- | --- | --- |
| 4.02  3.85 | 67.57 | A6 |
| 3.80  3.64 | 60.00 | B6 |
| 3.40  3.30 | 64.16 | 23 |
| 2.09  1.64 | 23.90 | 16 |
| 1.93  0.89 | 46.10 | 1 |
| 1.91 | 23.00 | 11 |
| 1.68  1.47 | 36.50 | 22 |
| 1.63  1.07 | 27.80 | 15 |
| 1.48  1.20 | 32.10 | 7 |
| 1.47  1.31 | 30.00 | 21 |
| 1.33 | 17.70 | 6 |

**-CH_3_:**

| δH | ^13^C |  |
| --- | --- | --- |
| 1.20 | 16.59 | R6 |
| 1.06 | 22.89 | 27 |
| 0.98 | 16.63 | 25 |
| 0.90 | 20.46 | 30 |
| 0.83 | 16.59 | 29 |
| 0.70 | 16.87 | 26 |
| 0.62 | 12.84 | 24 |

1. **Predicted binding modes**

3D representation of each compound in the HuR interaction site. HuR is shown as orange cartoon.


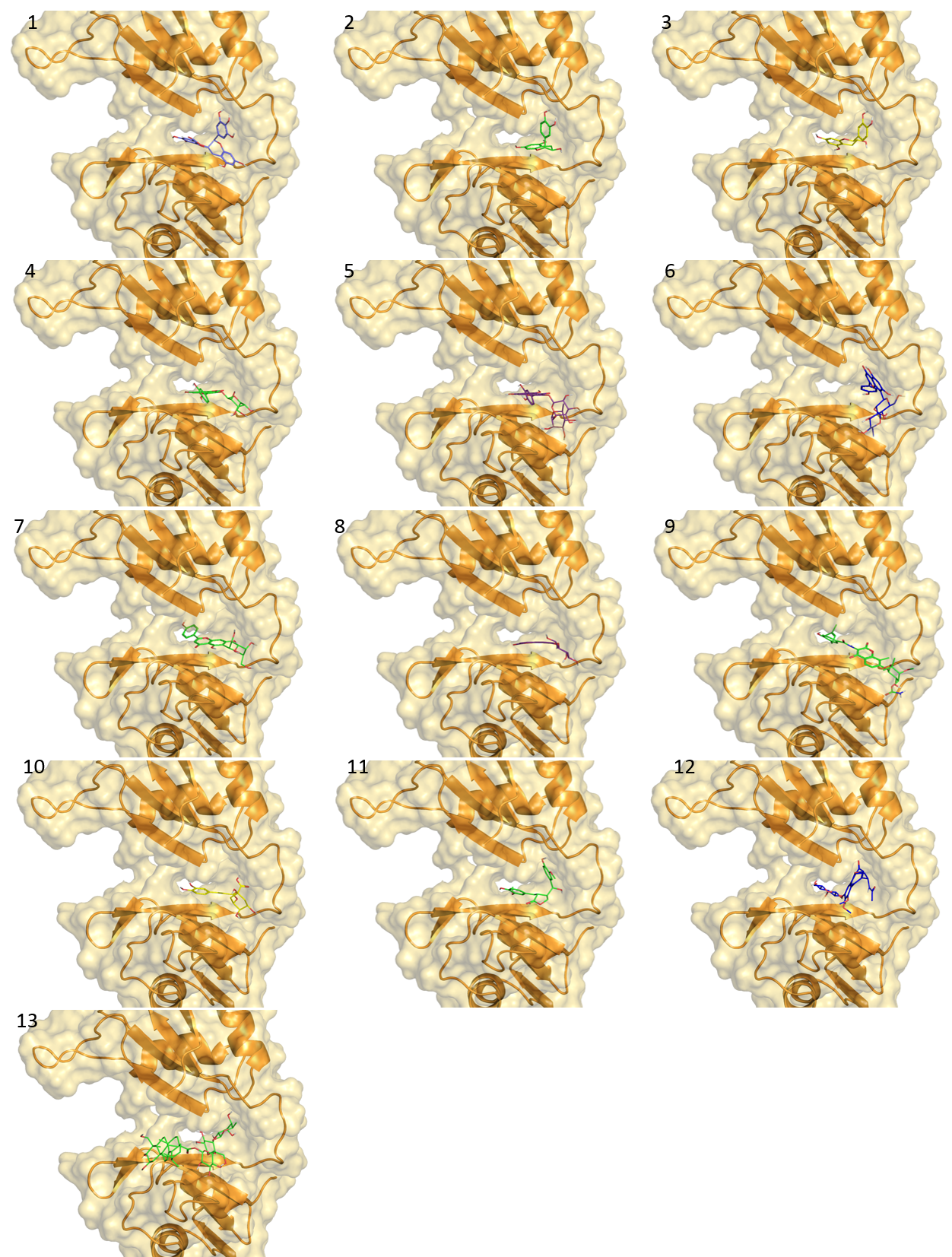


1. **Combined STD-NMR-Docking histograms for all interacting compounds**

The following histograms plot the number of interactions and related HuR residue (as seen through molecular modeling) and absolute STD% for each interacting proton (as determined by STD-NMR).

* values over 1%: 2’= 2.3%; 6’=1.5%; 6’’’= 2.7%

* values over 1%: 8-CH_3_= 1.1%; 13=1.2%

* values over 1%: 7’= 3.6%

* values over 1%: 8= 1.4%
